# Clinical and Drug Resistance Characteristics of *Providencia* Infections

**DOI:** 10.3390/microorganisms12102085

**Published:** 2024-10-18

**Authors:** Meenal Malviya, Pramodini Kale-Pradhan, Meredith Coyle, Christopher Giuliano, Leonard B. Johnson

**Affiliations:** 1 Division of Infectious Diseases, Henry Ford St. John Hospital, 22101 Morsoss Road, Detroit, MI 48236, USA; 2 Department of Pharmacy Practice, Eugene Applebaum College of Pharmacy and Health Sciences, Wayne State University, Detroit, MI 48201, USAek2397@wayne.edu (C.G.); 3 Henry Ford St. John Hospital, Detroit, MI 48236, USA; 4 Division of Infectious Diseases, Department of Internal Medicine, Henry Ford St. John Hospital, 22101 Moross Road, Detroit, MI 48236, USA; 5 Infection Prevention and Antimicrobial Stewardship, Ascension Michigan 22101 Morsoss Road, Detroit, MI 48236, USA; 6 School of Medicine, Wayne State University, 540 E Canfield St, Detroit, MI 48201, USA

**Keywords:** *Providencia*, resistance, outcomes

## Abstract

**Background:** *Providencia* is a G ram-negative bacillus that most frequently colonizes the urinary tract and is often resistant to many antimicrobials. This study aimed to evaluate the resistance patterns of *Providencia* spp. and clinical outcomes due to the paucity of data. **Methods:** A multi-center, descriptive, retrospective chart review of adult patients with *Providencia* spp. infections was conducted from 1 January 2020 to 31 May 2022. The primary outcome was to describe the drug resistance patterns of *Providencia* spp. isolates. This study’s secondary outcome was to evaluate the clinical outcomes of patients with *Providencia* spp. infections. **Results:** Of the 312 patients screened, 244 were excluded primarily for polymicrobial infections. The mean age was 70 years, and 39 (56.5%) were males. Of the 68 included cases, 46 (67.6%) were *P. stuartii*, 20 (29.4%) were *P. rettgeri*, and 2 (2.9%) were *P. alcalifaciens*. The most common infections were bacteremia 38 (55.8%), followed by 27 (39.7%) urinary tract infections and 3 (4.4%) wound infections. In this study, 45 patients (65.2%) had urinary catheters. The primary antibiotics used for treatment consisted of ceftriaxone (25 (36.2%)), cefepime (20 (29%)), and meropenem (10 (14.5%)). Only 5 of 68 (7.2%) cases were multidrug- resistant and required meropenem. In total, 19 patients (27.1%) died during their admission, but none were related to Providencia infections. A total of 10 of the 68 patients (14.5%) were readmitted within 30 days for reasons unrelated to the progression or recurrence of Providencia infections. **Conclusions:** *Providencia* bacteremia is predominantly seen in elderly patients. Third- generation cephalosporins remain an appropriate choice of antibiotics for *Providencia* spp. *Providencia stuartii* was the only species with multidrug resistance.

## 1. Introduction

*Providencia* is a G ram-negative bacillus in the Enterobacteriaceae family and includes five species: *Providencia stuartii*, *P. rettgeri*, *P. alcalifaciens*, *P. heimbachae*, and *P. rustigianii* [1]. These organisms are typically found in wounds, the respiratory tract, the urinary tract, the perineum, the axilla, and human blood and feces [2].

Generally, *Providencia* spp. are frequently resistant to tetracyclines, penicillins, and first- and second- generation cephalosporins. *Providencia* spp. may be susceptible to third- and fourth-generation cephalosporins, aztreonam, imipenem, and meropenem. *Providencia* species have variable susceptibilities to fluoroquinolones, aminoglycosides, and trimethoprim– sulfamethoxazole (TMP-SMX) [3]. Amikacin and beta-lactam/beta-lactamase inhibitors, such as piperacillin/tazobactam, are effective first-line agents in non- life-threatening infections [4]. *Providencia* spp. are intrinsically resistant to polymyxins and tigecycline, which are considered last- resort antibiotics for resistant pathogens [5]. The susceptibility to ciprofloxacin also decreased from 100% to 46% over a 6-year period (1986–1993) [6]. *Providencia stuartii* is typically the most resistant of all *Providencia* species. Limited antimicrobial options and the possible rapid progression of sepsis highlight the need for early detection in patients with *Providencia* bacteremia. There is limited literature describing *P. stuartii* infections [7,8,9], and there are no studies on *P. rettgeri* infections in the US. We evaluated the clinical characteristics and resistance patterns of *Providencia* Spp. infections. The primary outcome of this study was to determine the drug resistance pattern of *Providencia* spp. isolates. The secondary outcome of this study was to evaluate the range of infections and clinical outcomes of patients with *Providencia* spp. infections.

## 2. Methods and Materials

We conducted a multicenter, retrospective, observational descriptive study at the Ascension Michigan health system to determine the pattern of drug resistance of *Providencia* isolates, along with the clinical characteristics and outcomes of the patients. Five different hospitals of Ascension Health Southeast Michigan (Ascension St. John, Ascension Providence– Southfield, Ascension Providence– Novi, Ascension Macomb– Oakland, Warren, and Ascension Macomb– Oakland, Madison Heights). Data were collected from the electronic medical records. All patients with microbiology results reported as *Providencia* spp. from 1 January 2020 to 31 May 2022 were included in the analysis. Any patient with no microbiological results for *Providencia* spp. during the study period and those with polymicrobial infections were excluded. We followed the 31st edition of the Clinical and Laboratory Standards Institute (CLSI) guidelines. Some of the antibiotic susceptibility testing results were not released as part of the hospital protocol that includes carbapenem if cefepime is sensitive.

Multiple- drug resistance was defined as non-susceptibility to at least one agent in three or more antimicrobial categories. Extensive drug resistance was defined as non-susceptibility to at least one agent in all but two or fewer antimicrobial categories [10].

The primary outcome was to describe the drug resistance patterns of *Providencia* spp. isolates. The secondary outcomes included describing the antibiotics utilized, the duration of antibiotics, the type of infection, mortality, and 30-day readmission. In addition, mortality and readmission were evaluated to determine if the outcome was attributable to *Providencia* spp. Descriptive statistics were calculated to characterize the study group. Continuous variables were summarized using the mean value with standard deviation or median with interquartile range, and categorical variables will be presented as frequency distributions. The data collection included patient demographics, patient’s admission location (ICU versus general medical floor), infection- associated data including all cultures, susceptibilities, microbiological results, the location of the acquisition of infection (home versus healthcare), the presence of a urinary catheter, and other pertinent laboratory information (including serum creatinine, serum potassium, WBCs, etc.). The data collection on medications included antibiotic agent use during hospitalization and upon discharge and adverse reactions. This study was approved by the Ascension Institutional Review Board on 1 July 2022, and due to its retrospective nature, we did not obtain informed consent.

## 3. Results

Of the 312 patients screened, 244 were excluded primarily for the presence of a polymicrobial infection. The mean age was 70 years, and 39 (56.5%) were males. Of the 68 included cases, 46 (67.6%) were *P. stuartii*, 20 (29.4%) were *P. rettgeri*, and 2 (2.9%) were *P. alcalifaciens*. The majority of the patients were admitted to the general medical floor (81%), and 13 (19%) were admitted to the intensive care unit (ICU).

The most common infections were bacteremia (38 (55.1%)), followed by 28 (40.6%) urinary tract and 3 (4.3%) wound infections. In total, 45 patients (65.2%) had urinary catheters. The source of bacteremia could not be determined in every case due to the retrospective nature of this study. The primary antibiotics used for treatment consisted of ceftriaxone (n = 25 (36.2%)), cefepime (n = 20 (29%)), and meropenem (n = 10 (14.5%)). In total, 19 patients (27.1%) died during their admission, but none were related to *Providencia* infections. In total, 10 of the 68 patients (14.5%) were readmitted within 30 days for reasons unrelated to the progression or recurrence of *Providencia* infections. The treatment duration was 5.7 days with a standard deviation of 3.3 days.

### Drug Susceptibility Pattern

The resistance patterns of each of the three species of *Providencia* against different antibiotics including ampicillin, ampicillin/sulbactam, cefazolin, ceftriaxone, cefepime, Cipro, meropenem, nitrofurantoin, piperacillin–tazobactam, trimethoprim– sulfamethoxazole, gentamicin, tobramycin, and amikacin were reviewed.

*Providencia stuartii* species (46 isolates) were all resistant to cefazolin. Most of these isolates (35/46 (76.1%)) were susceptible to ceftriaxone and cefepime (42/46 (91.3%)). Table 1 depicts the MIC values of the various antibiotics for the different *Providencia* species. *Providencia stuarti* had high rates of resistance to fluoroquinolones and TMP-SMX. *Providencia rettgeri* had high rates of susceptibility to ceftriaxone, cefepime, ciprofloxacin, and TMP-SMX compared with *P. stuartii*. There were no extended- spectrum beta-lactamases (ESBL)-positive or carbapenem-resistant isolates identified. Ceftriaxone resistance can be a surrogate marker for ESBL production, but our ceftriaxone isolates did not have ESBL production.

## 4. Discussion

*Providencia* is a genus within the Enterobacteriaceae family closely related to the *Providencia* and *Morganella* genera. In the *Providencia* genus, *Providencia stuartii*, *Providencia rettgeri*, *and Providencia alcalifaciens* are the three species well- known to cause infections in humans, and among these, *P. stuartii* is the most frequently encountered human pathogen within the genus and was also most commonly encountered in our study. The most common site of isolation of *P. stuartii* is the urinary tract with long- term indwelling Foley catheters [2,10]. *P. rettgeri* can also be associated with nosocomial UTIs [2]. *P. alcalifaciens* is more common in waste water and soil reservoirs and is mainly associated with gastrointestinal illness, as reported in some studies [11,12]. One of the two isolates of *P. alcalifaciens* in our study was from a urine source and the other was from a wound.

*Providencia* species have the mannose-resistant/*Klebsiella*-like hemagglutinatin, also known as MR/K fimbriae, which gives the ability to stick to the urinary bladder as well as urinary catheters [13,14,15]. In our study, we had similar results with *Providencia*, which was most commonly isolated from blood and then urine. The next common site of infection after urine was wound infection. As with prior studies, *P. stuartii* was the most frequently identified species in our study.

We had no cases of mortality or readmission that were related to *Providencia* infections. The mean age of patients in this study was 70, which suggests that we had older patients, and perhaps our population may have a higher likelihood of colonization with hospital-acquired organisms, either because of repeated hospitalizations or coming from nursing homes. The duration of treatment was up to 14 days, which may be expected in some cases of bacteremia.

There are very few studies describing the susceptibility patterns of *Providencia* species. One single- center retrospective study from China found a high burden of extended-spectrum β-lactamases (ESBL)-positive *Providencia* species [9].

Virtually all *Providencia* species can produce inducible AmpC β-lactamases, and many isolates may also produce ESBL in nosocomial settings [16]. A 2006 Italian study found that ESBL-positive *P stuartii* made up 10% of all ESBL species and had marked resistance to amoxicillin–clavulanate (81.8%), ampicillin–sulbactam (40.1%), gentamicin (79.5%), and ciprofloxacin (84.1%) [4]. In another study, 53% of *P. stuartii* isolated strains were found to produce ESBL [17]. *P. alcalifaciens* and *P. rustigianii* tend to be the most susceptible of the *Providencia* spp. *P. rettgeri* tends to fall between the two groups mentioned above with regard to its susceptibility profile [3]. Another study from Portugal of approximately 300 isolates of *Providencia* species from different sources between 2000 and 2009 found over 90% of isolates were resistant to aminoglycosides (amikacin, gentamicin, isepamicin, and netilmicin), first-generation cephalosporins, and amoxicillin [18]. However, 99% of isolates remained susceptible to carbapenems (imipenem), third-generation cephalosporins (ceftriaxone, ceftazidime, and ceftibuten), and fourth-generation cephalosporins (cefepime).

Most isolates were susceptible to ceftriaxone and cefepime. Ceftriaxone resistance was mostly noted in *P. stuartii* compared with *P. rettgeri* and *P. alcalifaciens*. There were high rates of resistance to ciprofloxacin and TMP-SMX among *P. stuartii* isolates, but other *Providencia* species were highly susceptible to these agents. Cefepime and meropenem susceptibility testing results were not released if ceftriaxone was sensitive to avoid unnecessary and excessive use of them in the presence of more susceptible narrow- spectrum antibiotics like third-generation cephalosporin. We did not observe any CRE or ESBL isolates in our study. We followed the 31st edition of the Clinical and Laboratory Standards Institute (CLSI) [19,20,21] guidelines. Some of the antibiotic susceptibility testing results were not released as part of the hospital protocol.

There were several limitations to this study. This study had a small sample size, was retrospective in nature, and did not include polymicrobial infections. There was information missing on the exact discharge duration of antibiotics in some patients. Some of the antibiotic susceptibility testing results were not released due to the microbiology department policy. For example, our laboratory did not report aminoglycoside susceptibility results. However, most of the aminoglycosides are considered intrinsically resistant to *Providencia* species [3].

## 5. Conclusions

*Providencia* species, namely, *P. stuartii*, *P. rettgeri*, and *P. alcaligenes* can cause serious infections in elderly patients, with the most frequent being bloodstream infections. Third- or fourth- generation cephalosporins should be considered for empiric treatment instead of first- generation cephalosporins and fluoroquinolones. In our population, there were no ESBL and CRE isolates. There were no deaths or readmissions due to *Providencia* infections. Further studies should be conducted to confirm our observations.

## Figures and Tables

**Table 1:** Susceptibility of *Providencia* spp. against different antimicrobial agents.

	*Providencia stuartii*			*Providencia rettgeri*			*Providence alcalifaciens*		
	(n = 46)			(n = 20)			(n = 2)		
Antibiotic	S	I	R	S	I	R	S	I	R
Ampicillin-sulbactam	16	11	19	8	7	5	2	-	-
Ceftriaxone	35	-	11	18	-	2	2	-	-
Cefepime	42	2	2	19	1	-	2	-	-
Ciprofloxacin	7	1	38	19	1	-	2	-	-
Piperacillin-Tazobactam	42	-	4	18	-	2	2	-	-
Trimethoprim-sulfamethoxazole	26	-	20	17	-	3	2	-	-

S—sensitive. I—Intermediate sensitive. R—resistant. Ampicillin is intrinsically resistant for *Providencia*. Note—Carbapenem sensitivity was not released if cefepime was sensitive. None of the isolates had carbapenem resistance.

## Data Availability

Data unavailable due to privacy or ethical restrictions.
